# IMPLICATIONS OF GLOBAL CLIMATE CHANGE FOR THE ASSESSMENT AND MANAGEMENT OF HUMAN HEALTH RISKS OF CHEMICALS IN THE NATURAL ENVIRONMENT

**DOI:** 10.1002/etc.2046

**Published:** 2013-01

**Authors:** John M Balbus, Alistair BA Boxall, Richard A Fenske, Thomas E McKone, Lauren Zeise

**Affiliations:** † National Institute of Environmental Health SciencesBethesda, Maryland, USA; ‡ Environment Department, University of YorkHeslington, York, UK; § School of Public Health, University of WashingtonSeattle, Washington, USA; ‖ Lawrence Berkeley National LaboratoryBerkeley, California, USA; # Office of Environmental Health Hazard Assessment, California Environmental Protection AgencySacramento, California, USA

**Keywords:** Toxicology, Exposure pathway, Risk assessment, Vulnerability

## Abstract

Global climate change (GCC) is likely to alter the degree of human exposure to pollutants and the response of human populations to these exposures, meaning that risks of pollutants could change in the future. The present study, therefore, explores how GCC might affect the different steps in the pathway from a chemical source in the environment through to impacts on human health and evaluates the implications for existing risk-assessment and management practices. In certain parts of the world, GCC is predicted to increase the level of exposure of many environmental pollutants due to direct and indirect effects on the use patterns and transport and fate of chemicals. Changes in human behavior will also affect how humans come into contact with contaminated air, water, and food. Dietary changes, psychosocial stress, and coexposure to stressors such as high temperatures are likely to increase the vulnerability of humans to chemicals. These changes are likely to have significant implications for current practices for chemical assessment. Assumptions used in current exposure-assessment models may no longer apply, and existing monitoring methods may not be robust enough to detect adverse episodic changes in exposures. Organizations responsible for the assessment and management of health risks of chemicals therefore need to be more proactive and consider the implications of GCC for their procedures and processes. Environ. Toxicol. Chem. 2013;32:62–78. © 2012 SETAC

## INTRODUCTION

Global climate change (GCC) is associated with significant changes in long-term weather characteristics and short-term weather extremes in different regions. The world is becoming warmer overall, with increases in temperature being greatest over land and at high northern latitudes, and least over the Southern Ocean and northern North Atlantic. Snow-cover area is contracting and sea and mountain ice shrinking. Precipitation has increased in many regions at higher latitudes, while decreases have been observed in most subtropical land regions. These trends are expected to continue and intensify into the foreseeable future. While flooding rains are expected to become more common at higher latitudes, many areas that are currently semiarid are projected to experience more prolonged periods of drought. Future tropical cyclones are likely to become more intense, while extratropical storm tracks are projected to move toward the poles, changing wind, precipitation, and temperature patterns 1.

Chemical contaminants in the environment affect human health both directly and indirectly. Direct toxic effects range from acute poisonings and triggering of acute events like cardiac arrhythmias and asthma attacks to chronic effects like cancer and immunosuppression 2–4. Indirect effects include changes in health risks associated with changes in food supply and water sources as a result of chemical contamination or due to the selection of antibiotic resistance traits in bacteria exposed to veterinary and human antibiotics, metals, and other toxic substances 5–8. Because the persistence and mobility of toxic chemicals in the environment are affected by weather conditions such as temperature, precipitation, and wind, changes in these processes associated with GCC have implications for human exposures. Also, GCC is predicted to affect human diseases, change behavioral patterns that could influence exposures, and create additional physiologic stress through extreme temperatures. These human impacts of GCC, in addition to affecting health directly, may affect the vulnerability of humans to health risks from chemical exposures 9. Together, these changes in exposure and vulnerability to toxic chemicals may significantly alter human health risks.

To protect health, it is important that policy makers and regulatory organizations consider how GCC may influence chemical risks to humans and develop approaches to adequately assess and manage that risk. At the same time, in addition to GCC, there are other important future drivers of chemical risks, such as urbanization, demographic change, and developments in technology. These nonclimate drivers may also have positive or negative effects on exposure; and in some cases, they may have a bigger impact on human exposure than GCC alone. Future projections of potential chemical risks will thus need to integrate climate and nonclimate drivers.

The present study, one of a series arising from a SETAC-sponsored workshop to explore the potential influence of GCC on the foundation and applications of environmental toxicology and chemistry 10–15, presents options for addressing GCC in the assessment and management of the risks of chemicals in the natural environment to human health. Because this topic has not been a focus of the environmental toxicology community, there are few data to support a quantitative analysis of how specific chemical risks may be altered by GCC. Therefore, we begin by exploring the mechanisms by which GCC may alter human exposures and vulnerability to toxic chemicals. Using four specific examples of decision contexts for chemical risks, we then evaluate the robustness of current practices for chemical risk-assessment and management practices in the light of potential changes in human exposure and vulnerability caused by GCC. Where appropriate, we provide recommendations on how existing risk-assessment and chemical management practices could be improved to account for changes in exposure and vulnerability.

## ANTICIPATED CHANGES IN THE SOURCE–PATHWAY–RECEPTOR RELATIONSHIP

**Fig. 1:**
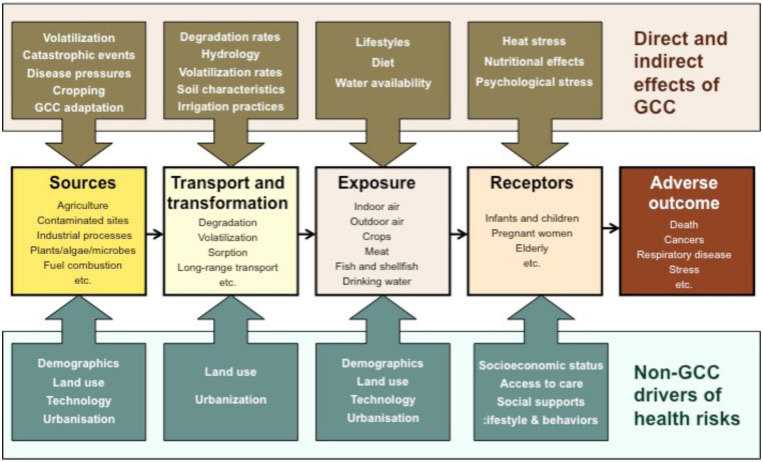
Source–pathway–receptor relationship, showing interactions with both climate and nonclimate stressors. GCC = global climate change. [Color figure can be seen in the online version of this article, available at http://wileyonlinelibrary.com]

For humans to be affected directly, chemicals must move through a pathway often called the source-to-receptor pathway. After being released from some source and moving through the environment, often being transformed by physical or biological factors in the process, chemicals must come into contact with and enter the body of a human. Once in the body, chemicals are subject to human toxicokinetic processes, ultimately coming in contact with some target tissue or molecular receptor to initiate an adverse outcome (Fig. 1). This chain of events is often complex, involving transformation of chemicals in the environment and their uptake and accumulation in organisms that play a role in exposing humans to those chemicals (e.g., consumption of contaminated fish). Changes in climate and associated changes in weather patterns, as well as non-climate-related drivers, are anticipated to affect various points in the source-to-receptor pathway, modifying how and to what extent humans are exposed to toxic chemicals and how they respond to harmful effects from those exposures 16.

### GCC-related alterations in chemical sources

Adaptation to GCC can influence the use and release of chemicals into the environment. For example, the types of pesticides, pharmaceuticals, and veterinary medicines used and the timing and frequency of their use will likely differ from today in response to changing disease and pest pressures resulting from GCC 17. Biocide use, for example, is likely to increase in response to increases in animal and plant pests and diseases that may arise from increasing temperature and humidity 17, 18. Expected decreases in fossil fuel use resulting from greenhouse gas mitigation policies will likely reduce ground-level air pollution by particulate matter and ozone in urban areas 19, while shifts in the production of some types of biofuels may increase levels of air pollution in rural areas; also, their use may increase their combustion product exposures 20. In addition to changing human patterns of chemical use and other behaviors, GCC may affect the rates of formation of natural toxins (such as fungal and algal toxins) in the environment as well as the geographical distribution of these substances 21, 22.

### GCC alterations in chemical fate and transport

It is also likely that GCC will combine with other natural and anthropogenic factors to affect the transport and transformation of toxic chemicals in the natural environment 11, 17, 23, 24. Increases in temperature can result in increased volatilization of persistent organic chemicals (e.g., at contamination sources such as buildings and electrical equipment 25), thereby increasing amounts subject to long-range transport 24, 26. Increases in temperature and changes in moisture content are likely to alter the persistence of chemicals 17, 23. Alterations in soil characteristics (organic carbon, dustiness) and hydrology may change how contaminants are transported around a terrestrial ecosystem as well as the dilution potential of contaminants in rivers and streams. Increases in the occurrence of extreme weather events, such as floods and droughts, will likely alter the mobility of contaminants. For example, flood events have been shown to transport dioxins, metals, and hydrocarbons from contaminated areas to noncontaminated areas 27, 28. In agricultural areas, changes in irrigation practices in response to GCC could also move contaminants from water bodies onto land 18. Changes in the degree and duration of ice cover may affect the degradation of contaminants in some regions 29. For legacy contaminants, such as mercury, that have been released to the environment in the past and reside in soil and sediments, GCC may alter the environment in such a way that the substances are remobilized or released more rapidly.

Increased temperatures can increase metabolic rates for many organisms, thereby increasing the potential for bioaccumulation and biomagnification of some contaminants 30. Temperature-related increases in the uptake and bioaccumulation of metals have been reported for several marine organisms, including crustaceans, echinoderms, and mollusks 21. In a recent modeling exercise of the uptake of methylmercury, increases in temperature resulted in increased concentrations of the compound in fish and mammals 31; temperature increases would also be expected to accelerate the conversion of mercury to methylmercury 32. In some instances, uptake may decrease. Studies of organochlorine concentrations in fish between 1994 and 2008 showed a decline in concentrations with increasing temperature, a trend that could be explained by declines in lipid content over time 33. The reason for the change in lipid content and a possible association with climate effects are unclear 33.

### Effects of GCC on human exposure to chemicals

Changes in the sources, fate, and transport of chemicals will have both positive and negative implications for contamination of food, drinking water supplies, air, and, hence, human exposure 16. However, very few studies have been performed to quantify the likely changes in exposure concentrations. In a U.K. study, Beulke et al. 34 quantified the effects of changes in the use, fate, and transport of pesticides, resulting either directly or indirectly from GCC, on surface water and groundwater concentrations. The study showed that concentrations of pesticides in surface waters and groundwaters are likely to increase under GCC and that, for some pesticides, peak concentrations could increase by orders of magnitude. The indirect effects of GCC (i.e., effects on amount of pesticide applied and application timing) on surface-water exposure were found to be stronger than the impact of changes in the climate alone on chemical fate and transport. Additional studies of this type are needed for other classes of pollutant and other geographical regions.

Global climate change will affect not only the concentrations of toxic chemicals in water, food, and air, but also how humans interact with these media; this will also have implications for the degree of human exposure. For example, reduction in the availability of drinking water for many populations could change exposure to waterborne contaminants as human populations shift toward other sources of drinking water (e.g., water from water-reuse and reclamation systems) 35. Changes in climate may also impact the amounts of time humans spend indoors and outdoors, influencing exposure to both indoor and outdoor contaminants 36.

As very few robust studies into the effects of GCC on human exposure to chemicals are available, the authors have attempted to develop a qualitative assessment of how GCC is likely to affect the sources and distribution of a variety of pollutants with an assessment of the likely impact on human exposures (Table 1). Table 1 illustrates that the effects of GCC are likely to be very situation-, chemical class-, and source-specific, and will vary across different geographies and at different times of the year.

**Table 1:** Potential effects of global climate change (GCC) on human exposures from major chemical contaminant sources

Contaminant of concern	Relative importance of environmental exposure pathways compared to other pathways (e.g., direct use, occupational)	Major sources	Direct and indirect effects of GCC on contaminant source	Direct and indirect effects of GCC on fate and transport	Overall effect of GCC on human environmental exposure	Evidence base for climate impact
Criteria air pollutants SO_2_, NO_x_, PM_x_	High	Automobiles, chemical production sites, power stations, incinerators, residences	Emissions of many air pollutants harmful to human health may be lower if society mitigates against greenhouse gas emissions by moving toward biofuels 72. Emissions may increase at biofuel production sites 20.	Limited	Adverse/positive	High
Ground-level O_3_	High	Precursor gases NO_x_, VOCs, and CO	If global carbon emission controls are implemented, a reduction in ozone is predicted in developed areas, but an increase is expected in developing countries. If controls are not implemented, exposure will increase globally. An increase expected in number of pollution days is also expected 81.	Limited	Adverse/positive	High
Arsenic	High	Natural contamination	Altered climates that impact the microbial communities and water chemistry of freshwater systems can affect the distribution of arsenic species in surface water 82.	Increased irrigation results in greater contamination of food crops 83.	Adverse	High
Mercury	High	Contaminated soils and sediments	Altered climates may affect microbial biotransformation of mercury from the divalent to the more biologically available organic species 33.	Increases in temperature may increase Hg mobility as a result of increased conversion of Hg species to vapor Hg and to methyl mercury 80. Uptake into fish expected to increase 33.	Adverse	High
Dioxins, PCBs, DDT	High	Waste sites, combustion, electrical equipment, forest fires	Increasing temperatures will increase the release of POPs from sources such as buildings and electrical equipment 27. Use of DDT may increase due to increased need for malaria control 27.	Thawing of ice caps will release persistent organic compounds. Increases in flooding events will remobilize soil- and sediment-associated POPs and transport them to uncontaminated areas 18. Uptake into fish may increase or decrease 84. Degradation of some POPs will increase in some areas 85	Adverse	High
Pesticides	Medium	Agriculture	Outbreaks of a wider variety of insects and pathogens is expected, resulting in increased pesticide use and changes in application timings 58. Geographical shifts in agriculture will mean that cropping patterns change and types of pesticide use change 86.	Increased volatilization of pesticides expected following application 1. Degradation in soils may increase due to increased temperatures and changes in soil moisture 25.	Adverse—bystander exposure anticipated to increase	Medium
Pharmaceuticals	Low	Health care	Increases in human diseases (e.g., malaria, depression) may result in increasing pharmaceutical use.	Increased recycling of water and use of treated wastewater for irrigation may result in entry into food items.	Adverse	Low
Veterinary drugs	Low	Agriculture	The proliferation of animal diseases due to climate-related changes may result in an increased use of veterinary drugs that could lead to increased releases to the environment 18.	Degradation in soils may increase due to increased temperatures and changes in soil moisture 25.	Adverse	Medium
Industrial process chemicals	Low	Industrial facilities	Increased catastrophic weather events (floods, wildfires) may result in increased catastrophic accidental releases.	Limited	Adverse	Low
Algal toxins	High	Algal blooms	Warmer conditions are generally linked to increased frequency, duration, and geographic scope of HABs, due to changes in water temperatures and stratification and increased nutrient inputs, so GCC is predicted to increase the incidence of HABs in the future 18, 23, 86, 87	Limited	Adverse	High
Mycotoxins	High	Fungi-infected crops	Mycotoxin contamination of crops is anticipated to increase due to drought stress, temperature stress, stress induced by pest attack, increases in arthropod vectors, poor nutrient status 54, 86. Changes in the geographical range of crops produced could provide opportunity for new fungus–plant associations to arise, so mycotoxins currently not considered a threat to public health (e.g., sterigmatocystin, cyclopiazonic acid, moniliformin) may become important 18.	Limited	Adverse	High
Pollen	High	Plants	GCC is predicted to lengthen the pollen season as well as increase pollen and spore rupture; increase in pollen allergenicity 22, 88	Increase in long-distance transport	Adverse	High

SO_2_ = sulfur dioxide; NO_x_ = nitrogen oxide; PM_x_= particulate matter; O_3_ = ozone; VOC = volatile organic compound; CO = carbon monoxide; Hg = mercury; POP = persistent organic pollutant; PCB = polychlorinated biphenyl; DDT = dichlorodiphenyltrichloroethane; HAB = harmful algal blooms

### Impacts of GCC on human vulnerability and risk

The analysis in Table 1 indicates that while climate change could have a positive effect on exposure, in most instances an adverse effect is anticipated in certain regions and it is possible that this will result in adverse health outcomes. Table 2 provides examples of various health effects and risks for some of the chemical exposures likely to be impacted by GCC. It shows GCC affecting the incidence and episodic frequency of acute events, such as cardiovascular and respiratory mortality from high–air pollution episodes. In regions of some developing countries, the increase in the extent and magnitude of episodes of algal and fungal infestations is likely to correspond to increasing mycotoxin exposures, with attendant increasing occurrence of target organ toxicity and cancer, especially in areas where fungal infestations are endemic and populations are vulnerable 18, 24, 37. For a number of the persistent organic pollutants, with the increased exposure expected in some regions comes increased risk of cancer and endocrine, neurological, and reproductive toxicities (38, see Table 2).

**Table 2:** Health effects and vulnerable populations for selected environmental exposures potentially affected by climate change

Stressors	Health effects	Possible most heavily impacted populations or regions	Confidence in or evidence for effects
Mycotoxin residues in foods (effects indicated are for aflatoxins)	Carcinogenicity, hepatotoxicity, immunosuppression, developmental toxicity 89, 90, and possible male fertility deficits	Developing areas of Africa, Asia, and South America with high concentrations of relatively uncontrolled mycotoxin exposure	Sufficient evidence for carcinogenicity in humans for aflatoxins 89 and in animals for fumonisins 91 and ochratoxin 92, 93. Suggestive evidence of reproductive toxicity in humans.
Algal toxins (entries in table are for the blue-green algae toxin microcystin-LR)	Severe gastroenteritis, liver toxicity, blistering of mucous membranes, possible immunotoxicity, possibly carcinogenic 94–96	Those recreating in eutrophic water that harbor algal cyanobacterial populations; people on water supplies from sources that harbor the bacteria but are not adequately treated (disinfection can be insufficient); greater consumption of contaminated fish, shellfish, and crayfish by those with lower income. Because they are stable to heat and acid, food preparation does not protect consumers of contaminated fish or shellfish.	Carcinogenicity conclusion of systematic IARC review 94; noncancer evidence from documented episodes of human poisoning as well as from animal experiments
Ozone	Asthma exacerbation, chronic pulmonary obstructive disorder, cardiovascular and pulmonary disease 97, 98	People with preexisting health conditions such as asthma; people in communities and developing countries with existent high concentrations of ozone; in US, urban centers in the mid-Atlantic and northeast.	Clear evidence from studies in humans
Methylmercury	Neurotoxicity 99	Fetus and young more impacted at same dose than adults; subsistence fishers	Clear evidence in humans of neurotoxicity in adults and the young
Polyhalogenated biphenyls, dioxins, furans, and other halogenated POPs	Carcinogenicity, endocrine toxicity, neurotoxicity, reproductive toxicity 89, 100, 101	Fetus and young more impacted by thyroid hormone–related toxicity pathways; susceptibility of native populations (e.g., Arctic)	Dioxin is a known human carcinogen 89. There is sufficient animal evidence supporting the potential carcinogenicity of many POPs 100. There is a large volume of evidence supporting endocrine toxicity for many POPs, such as PCBs 101.
Pollens	Asthma, allergic rhinitis 102–105	Children and older adults	Increase in temperature associated with increased pollen and increased percentages of patients sensitized to pollens have been observed, but good epidemiologic evidence is lacking 22.

GCC = global climate change; IARC = International Agency for Research on Cancer; POP = persistent organic pollutant; PCB = polychlorinated biphenyl; PM_x_ = particulate matter; SO_2_ = sulfur dioxide; VOC = volatile organic compound.

In addition to increased exposure to individual contaminants influencing the existing disease burden, nonchemical stressors related to GCC may alter the vulnerability of humans to toxic insults. The extreme temperatures 39, 40 and high ozone levels experienced during heat waves are independently associated with increased cardiovascular mortality, but temperature also appears to interact with ozone, increasing short-term mortality beyond that expected for either stressor acting independently 41–44. Temperature has also been observed to modulate the impact of cardiovascular mortality due to particulate matter in ambient air 45, 46.

There has been limited study of the effect of temperature on the toxicity of chemicals in animal models. Temperature has been observed to increase neurotoxicity from methamphetamine exposures in mice 47, and in general, increasing temperature exacerbates chemical toxicity in animal models 48. A number of other nonchemical stressors have been reported to modify chemical toxicity, such as certain pre-existing infectious and noninfectious diseases, nutritional status, and exposure to violence and other psychosocial stressors 49–51. Changes in food-chain structure will likely lead to alterations in the diet, with attendant changes in nutritional status and vulnerability 52. Psychosocial effects of GCC include social and community effects of heat such as violence 53, which may also heighten overall vulnerability to chemical exposures.

## IMPACTS OF GCC FOR CHEMICAL RISK ASSESSMENT AND MANAGEMENT

A number of chemical management processes are used in different regions of the world to protect human health from chemical exposure, including the setting of standards for chemical contaminant concentrations in food, air, and water and associated monitoring practices; the embargo or removal from the market of products posing unacceptable risks and related assessment methodologies; warning labels; the mitigation of site risks and related planning analyses; and the development of long-term national and international chemical strategies. Because the influence of GCC on chemical risk management may vary among these different situations, we explore in more depth the decision contexts associated with these processes. Chemical management typically takes into account both short-term and long-term risks, information on toxicity of specific substances, and behavioral and other population characteristics that influence exposure and vulnerability. For example, in approving a pesticide, regulatory agencies typically consider the consequences of acute occupational exposure in someone applying the pesticide, the longer-term fate and transport of that pesticide in the environment or food crop, and the extent to which susceptible populations, such as infants and children, are exposed. As another example, decisions for positioning and permitting of facilities for generating or storing hazardous substances typically take into account both exposure pathways related to the long-term release of chemicals and the probability of flooding, storms, or other weather extremes that may lead to sudden large releases and exposure. The relative importance of changes related to GCC for each of these decision contexts will vary based on the specific questions asked and decisions to be made, the time scale under consideration, and the characteristics specific to the geographic location and populations being analyzed.

In the following sections, four broad groups of chemical contaminants (natural toxins, pesticides, air pollutants, and legacy contaminants) are used to illustrate the implications of GCC for different chemical-assessment and/or management mechanisms.

### Natural toxins

Natural toxins are produced by algae (e.g., microcystins), bacteria (e.g., botulinum), plants (e.g., glycoalkaloids, anisatin), and fungi (e.g., aflatoxins, ochratoxins, zearalenone) and include some highly toxic chemicals. These compounds have been reported to elicit a range of effects on human health, including death, effects on growth, liver cancers, cirrhosis, gastrointestinal disease, neurotoxicity, and severe dermal toxicity 54–57. Production of these compounds can be very sensitive to environmental factors such as temperature and humidity 54. Indirect effects of GCC may also be important; for example, changes in the distribution and activity of insect vectors may increase the exposure and vulnerability of plants to mycotoxins 54, and increases of runoff of nutrients from agricultural systems and changes in dilution may increase the occurrence of algal blooms 17. Therefore, GCC is predicted to increase human exposure to phytotoxins and mycotoxins in some areas 58. As some of these substances are particularly potent in terms of toxicity to humans, these increases in exposure could have a significant negative effect on the health of populations in certain regions.

Human exposure to natural toxins is currently controlled in many countries through monitoring of concentrations of these compounds in crops, shellfish, and drinking water, although in some regions monitoring is nonexistent. Therefore, any effect of GCC on the occurrence and toxicity of natural toxins to humans will be of direct interest to regulatory and public-health agencies responsible for monitoring these compounds and to suppliers of food or drinking water. The potential impacts of GCC on the occurrence of natural toxins and subsequent impacts on food security in different regions will also be of interest to policy makers at the national and international levels. An analysis of the implications of GCC on the monitoring of natural toxins for food-safety purposes (Table 3) suggests that, due to the likely increase in occurrence, existing monitoring mechanisms may not be robust enough to protect human health in the future. Increases in the occurrence of natural toxins may also compromise the security of foodstuffs and drinking water in some regions, resulting in negative effects on health and well being. Fundamental research and programmatic questions include whether existing monitoring schemes are adequate to protect humans against exposure to natural toxins in the future and the likelihood and potential consequences of an increased incidence of natural toxins in food and water supplies.

**Table 3:** Impacts of global climate change (GCC) on different assessment or management processes for natural toxins

Decision question being asked	Who?	How is the decision made now?	What will be the implications of GCC on the decision-making process?	Probability and magnitude of GCC impact	What other changes might take place?	Recommendation
*Monitoring*						
Is the level of a natural toxin (e.g., mycotoxin or algal toxin) safe?	Regulatory and public-health agencies, food industry	Occurrence of some natural toxins is measured in food samples and concentrations are compared to a standard or advisory guideline.	Outbreaks of fungal infections and algal blooms will occur more frequently under GCC. More rigorous monitoring may be required to pick up exceedances.	High probability in some regions, high magnitude	None anticipated	Modeling studies to explore likely prevalence of natural toxin food contamination incidents in different regions and evaluation of existing monitoring regimes against these predictions.
*Strategic forecasting*						
Do natural toxins pose a threat to food security?	Regulatory and public-health agencies, public-health researchers	Analysis of monitoring data on the occurrence of natural toxins in food	Outbreaks of fungal infections and algal blooms will occur more frequently under GCC, resulting in reduction in the availability of safe food in some regions.	High probability, magnitude of changes uncertain	None anticipated	Apply plant infection models that account for climatic effects for different regions under GCC.
						Apply modeling to explore likely increases in algal blooms under GCC in different regions.

### Pesticides

Pesticides are important tools in much of modern agricultural production and are critical for vector control in many parts of the world (Table 4). It is anticipated that pesticide use will increase in many parts of the world in response to greater pest activity associated with increasing temperatures 17.

**Table 4:** Impacts of global climate change (GCC) on different assessment or management processes for pesticides

Decision question being asked	Who?	How is the question answered now?	What are the implications of GCC on the assessment or decision process?	Probability and magnitude of GCC impact	What other changes might take place?	Recommendation
*Standard setting*						
What is a safe concentration of a pesticide on food?	Regulatory and public-health agencies	Acceptable dose is determined from dose–response studies.	Increased vulnerability of population may require lower standard or guideline.	Medium, may be high for particular subpopulations	New research may change toxicological risk estimates	Toxicologic studies with climate costressors and adaptation of methods for estimating acceptable doses.
		Dietary consumption patterns are used with dose data to establish acceptable concentration standard or guideline.	GCC may impact food production and, hence, availability of certain food types, changing dietary patterns.	High probability, high magnitude for some regions; low/medium for others	Diets may change due to cost or nutritional needs; world economic crisis could occur, resulting in higher food prices; transportation of food may become more costly because of energy costs and carbon taxes.	Dietary consumption should be recalculated more frequently and for vulnerable regions and subpopulations.
*Monitoring*						
Is the concentration of pesticide in the food supply safe?	Regulatory and public-health agencies, food industry	Pesticide residues are measured in food samples, and concentrations are compared to tolerance standard or guideline	Pesticide applications may occur more frequently under GCC. More rigorous monitoring may be required to pick up exceedances.	Medium probability, medium magnitude	Safer product may be substituted for current pesticide; new pest-management techniques may be adopted; organic farming may become more widespread.	Modeling studies to explore likely changes in occurrence of pesticides in food under GCC in different regions and evaluation of existing monitoring regimes against these predictions.
*Product risk assessment*						
What will be the risk of a new pesticide to humans via food?	Regulatory and public-health agencies, pesticide registrants	Predict pesticide concentrations in food based on expected uses.	New pathways of exposure (e.g., dust) may occur that are not currently modeled;	Low; registrants may anticipate major GCC impacts through market demand studies.	Changes in market demand may result in dietary exposures that are different from those predicted.	Test food samples for residues on a regular basis.
			existing model algorithms may fall down under GCC extremes.			
		Predict exposure based on dietary information.	Diets may change under GCC.	High for some regions, low/medium for others	Economic factors may alter dietary choices.	Conduct periodic food-consumption surveys.
		Compare predicted exposures to toxicologic data.	Increased vulnerability may mean current assumptions in risk assessment are no longer valid.	Low/medium, high uncertainty	New scientific studies may alter risk-assessment inputs	Regularly review scientific literature on vulnerability under GCC
*Strategic forecasting*						
Will we be able to access food that is safe from pesticides in the future?	Regulatory and public-health agencies, public-health researchers	Periodic updates of risk assessments	Increased pesticide use and irrigation practices may increase residues in food. Increased UV may decrease residues.	High probability, magnitude of changes uncertain.	Economic factors may limit the ability to collect sufficient data for risk assessments.	Provide support for data collection, conduct reregistration reviews more frequently.
			Safe concentrations may change (see above).			

UV = ultraviolet.

Many insecticides are acutely toxic, and their use in agriculture has resulted in poisonings among workers 59, 60. The long-term health effects of pesticide exposures are less certain, but reviews have identified significant health outcomes in agrarian populations 61, 62. Efforts to minimize human exposure to pesticides have generally focused on the following three exposure settings: pesticide residues in food and drinking water; exposures in farmers, farmworkers, and applicators; and community exposures.

The case of pesticide residues in food is used to illustrate the decision context for risk assessment in the face of GCC. Table 4 indicates that many factors other than GCC might influence dietary pesticide exposure, and GCC could be of minor consequence in some instances. Also, GCC could result in reduced exposure and risk under certain conditions. However, in nearly all cases it is expected that GCC will add to the complexity of risk assessments. The significant changes in exposure to pesticide residues via food are likely to result from changes in decisions on the selection of particular pesticides for application.

Setting of pesticide residue–tolerance levels in foods is a key regulatory activity to protect the safety of the food supply. It requires dose–response data and information on the dietary habits of numerous subpopulations 63. We also foresee worker populations becoming more vulnerable to the effects of pesticides due to enhanced skin absorption from exposure to extreme heat, and we expect dietary habits to change based on food cost and availability. These changes will require more frequent updating of risk assessments. Similarly, monitoring for pesticide residues is needed to ensure a safe food supply and will need to be conducted more often as GCC alters pesticide use. New product registrations are based on regulatory risk assessments, and these are revisited periodically after a new chemical is in use. Table 4 shows how the underlying approaches to monitoring pesticides and assessing their risk may require re-evaluation in the context of GCC. The ability to forecast the safety of the food supply will become more uncertain with more rapid changes in pest-management and dietary behaviors.

### Air pollutants

Human activities, especially combustion of fossil fuels for energy, release a wide array of toxic substances into the air (Table 5). Natural processes, ranging from wildfires to biological processes of life and decay, also contribute to harmful air pollution. The two air pollutants associated with the greatest burden of death and disease are fine particulate matter (PM2.5), which comes from a variety of anthropogenic and natural sources, and ozone, which is mostly formed in the atmosphere from a chemical reaction between nitrogen oxides, sunlight, and volatile organic compounds. Health effects associated with both air pollutants include the exacerbation of chronic lung disease, asthma, and myocardial infarction. Particulate matter has also been associated with adverse birth outcomes and neurodevelopmental delays. In the United States, it has been estimated that fine particulate air pollution was responsible for over 130,000 deaths and ozone air pollution for over 4,700 deaths in the year 2005 64.

**Table 5:** Impacts of global climate change (GCC) on different assessment or management processes for air pollutants

Decision question being asked	Who?	How is the decision question answered now?	What will be the implications of GCC on the decision-making process?	Probability and magnitude of GCC impact	What other changes might take place?	Recommendation
*Standard setting*						
What is a reasonably safe concentration of a criteria air pollutant with respect to human health?	Regulatory and public-health agencies and scientific advisory entities, affected industries	Acceptable concentration is determined from meta-analysis of epidemiologic studies	GCC will have mixed impacts on primary and secondary pollutant levels; may affect population vulnerability through costressors (heat) or secondary effects (nutrition, stress); will affect validity of models used for regulatory justification.	High probability; magnitude of impact uncertain; small magnitude could have large public-health consequences for PM2.5 and ozone.	Changing technologies for energy generation and transportation; changing land-use and agricultural practices; GCC mitigation measures in general likely to reduce emissions of criteria air pollutants. This may be offset to a certain extent by population growth.	Research needs: (1) laboratory simulations to determine effects of temperature and other atmospheric parameters on secondary pollutants, especially particulates; (2) studies of interactions of weather parameters and pollutant exposures in humans; (3) controlled human clinical studies of temperature impact on pollutant toxicity.
						Practice needs: updating of weather models in regulatory justifications, ensuring that weather models reflect current and future trends in variability, extreme events, air stagnation, events, etc.
What is a reasonably safe concentration for criteria air pollutants with respect to human welfare?	Regulatory and public-health agencies	Acceptable concentration is based on consideration of a wide array of scientific literature on the effects of criteria air pollutants on ecosystems, crops, and other receptors linked to human economic well-being and indirectly to health	GCC is likely to alter the vulnerability of many ecosystems and plant species to criteria air pollutants (e.g., ozone) due to costressors, including pests and diseases.	High probability, likely high impact	Technological advances in genetically engineered crops to resist heat, drought, and pests. Changes in agricultural practices and recreational patterns.	Maintain and strengthen programs that evaluate ecosystem health; document changes due to climate, agricultural, and recreational patterns.
*Monitoring*						
What are the concentrations of air pollutants in critical geographical areas?	Regulatory, health, and resource-management agencies	Monitoring conducted primarily for regulatory compliance purposes, frequency and location determined by statutes or regulations, and guidance developed by stakeholder processes; monitoring for research conducted on an ad hoc basis.	Changing distribution of air pollutants may shift most important places for monitoring; GCC will require more frequent monitoring.	High probability, low to moderate impact	For developing countries, unclear to what extent satellite-based monitoring will be developed	Research needs: (1) for developing countries, improved satellite monitoring methods; improved, cheaper surface-based monitoring devices.
						Practice needs: expanded air-quality monitoring in developing countries
*Product risk assessment*						
What are the human health risks associated with fuel additives, biofuels, and other new technologies intended to reduce greenhouse gas emissions?	Regulatory agencies, producers	Laboratory testing of product and characterization of by-products of combustion; standard toxicological risk assessment based on modeled exposures and toxicity tests.	Primarily related as a technological intervention to address the problems of GCC; future GCC may affect use patterns as well as dispersion models used to estimate human exposures.	High probability, low impact	Technological developments not driven by GCC	Use realistic ranges for future use scenarios, avoiding historical errors of underestimates of market penetration and use; ensure dispersion and exposure models are robust for future climate parameters.
*Strategic forecasting*						
What policy options for reducing greenhouse gas emissions offer the greatest human health cobenefits through reduction of toxic criteria air pollutants?	Regulatory and public-health agencies, academic researchers, expert consultants, industry	Process involves individual policy analysis, primarily through NEPA; use of linked risk-assessment models like BenMAP or integrated assessment models	Feedback loops of GCC on factors in integrated assessments such as economic vitality, patterns of energy consumption and transportation use; impacts of altered climate on current dispersion and exposure models	High probability, uncertain impact	Concurrent changes in many factors, including economic, social, underlying health status, demographics, technologies	Research needs: improved integrated assessment models with improved modules for human health risk assessment

NEPA = National Environmental Policy Act; PM_x_, = particulate matter.

Concentrations of air pollutants in the atmosphere are strongly influenced by weather conditions. Air stagnation and especially air inversions lead to high concentrations of fine particulate matter, while precipitation and high winds tend to lower concentrations. Ozone concentrations are highest on days with high temperatures and high concentrations of ultraviolet radiation from sunlight. In addition to directly affecting concentrations of PM2.5 and ozone, weather and climate conditions indirectly affect concentrations through influences on natural sources of precursors to these pollutants and on human activities that result in emissions (e.g., increased energy consumption for heating and air conditioning). By altering prevailing patterns of temperature, precipitation, winds, and atmospheric penetration of sunlight, GCC is anticipated to lead to both increases and decreases in air pollutant concentrations 65, 66.

Human health risks from criteria air pollutants are managed by regulatory standards for ambient air concentrations and by emissions-control standards for specific sources, permitting requirements, and other measures. Decisions regarding regulatory measures rely on health risk assessments that in turn use observed concentrations and air-quality models to determine population exposures. Only a few countries around the world have established air-quality monitoring networks to provide accurate data on pollutant concentrations; new techniques, such as satellite observations, may play an important role in the future management of human health risks, especially in developing countries 67. Air-quality models include assumptions about weather conditions to determine critical model parameters that control air pollution–concentration estimates. As GCC progresses, these models will need to be revised to reflect changes in temperature and precipitation and the frequency of unusual weather events.

It is possible that GCC will also contribute to changes in population vulnerability to harmful air pollutants. For example, added cardiovascular stress from high summer temperatures may increase sensitivity to the cardiovascular effects of PM2.5 and ozone 44, 67, 68. Conversely, warmer winter temperatures may both reduce cardiovascular stress and lower emissions of combustion products because of reduced heating needs. Because both human and animal studies of air pollution have devoted few resources to the study of interactions between temperature and air pollution exposures, the extent of change in vulnerability related to higher temperatures is uncertain and comprises a research gap.

Many harmful air pollutants are created by the same processes that produce greenhouse gases, so significant measures to reduce greenhouse gas emissions will in many cases lead to reductions in harmful air pollutants as well. Several studies indicate substantial health cobenefits from these air-pollution reductions resulting from greenhouse gas–reduction measures 69–71. Estimates of these health benefits need to account for GCC-related influences on the processes that determine air-pollutant concentrations. Some measures to reduce greenhouse gas emissions, such as the development of alternative fuels and fuel additives, may create novel air pollutants or increase concentrations of existing harmful ones 72, 73. These changes in exposure and the consequent health effects must also be anticipated and assessed in making decisions about greenhouse gas reductions and air pollution.

### Legacy pollutants

Legacy pollutants are persistent substances that have accumulated in environmental reservoirs such as surface soils, ice, sediments, and forests 74–76. Their slow and continuous emissions from these reservoirs pose a long-term risk to human population and ecosystem health 77. Legacy pollutants include dioxins and dioxin-like compounds, PCBs, mercury released into the environment by mining and combustion processes, radioactive compounds from nuclear weapons testing, DDT, lindane, and others 75. The health effects of these pollutants range from cancer, adverse reproductive outcomes, and impaired neurodevelopment to disruption of the endocrine and immune systems. Unlike the other risks highlighted in the present study, the management of legacy pollutants, at least in developed countries, primarily involves tracking reservoir sources and mitigating exposure rather than controlling or reducing emissions from ongoing economic/industrial activities 78. Developing countries, in addition to often lacking effective monitoring and management of legacy pollutant waste sites, may have populations highly exposed by ongoing recycling and waste-processing activities 79.

Because legacy pollutants persist and bioaccumulate in the environment, longer-term environmental processes related to GCC could influence their fate and transport and alter human and ecosystem exposures 80. The transfer of legacy pollutants among environmental media determines their relative abundance in mobile (water or air) compartments versus reservoir compartments (soil and sediments). Legacy pollutants migrate from one region to another through advection in a mobile phase such as air or water 74. If GCC results in stronger winds and/or stronger river, lake, estuary, and ocean currents, regional and global migration patterns will be altered. The persistence of legacy pollutants depends on chemical transformation processes, some of which are climate-dependent. It will be necessary to understand the potential impacts of GCC on processes such as hydrolysis and biotransformation, which play a key role in removing chemicals from environmental media such as soil, water, and sediments (Table 6).

**Table 6:** Impacts of global climate change (GCC) on different assessment or management processes for legacy pollutants

Decision question being asked	Who?	How is the decision question now answered?	What will be the implications of GCC on the decision-making process?	Probability and magnitude of GCC impact	What other changes might take place?	Recommendation
*Monitoring*						
What are the concentrations of legacy pollutants in the environment and food supply?	Regional, national, and international regulatory and public-health agencies	Legacy pollutants such as mercury and legacy POPs are tracked in air, water, soil, food, and biota. There is also a strong reliance on fate modeling to interpret future trends and provide more spatial resolution.	Legacy pollutants can be more easily mobilized by climate disruption and can have altered phase distributions in warmer climates.	High probability of change in higher latitudes and at higher elevations. Moderate probability of change in other regions.	Higher-resolution environmental and biospecimen monitoring capacity	Expanded modeling studies combined with strategic environmental sampling to track the magnitude and variation of altered transport patterns for legacy pollutants, particularly in higher latitudes. Careful attention to food-web contamination is needed.
						
*Strategic forecasting*						
How safe are the levels of legacy pollutants in the food supply and breast milk?	Regional, national, and international regulatory and public-health agencies	Analysis of food concentrations compared to guideline levels established using data from toxicological tests and predicted food concentrations	Food commodities in some regions, particularly for subsistence populations, may need to be replaced or consumption curtailed; fish advisories and other public information programs likely will also be affected.	High probability of increased exposure, but significant geographic and population variability are likely.	Availability of seafood could be more substantially impacted by GCC or overfishing than anticipated.	Decision makers should exercise adaptive management to address changes, based on effective use of expanded models and limited observations. Models used to support selection of chemicals of concern for the Stockholm Convention on POPs should be reevaluated.

POP = persistent organic pollutant.

## CONCLUSIONS AND RECOMMENDATIONS

Global climate change may affect multiple steps in the process of human exposure and harm to human health from chemical risks, including emissions from sources, transport and transformation in the environment, and human behaviors and vulnerabilities. For many chemical contaminants, a net increase in exposure is likely in certain regions of the world. While GCC will affect the fate and transport of chemicals in multiple and complex ways, in many cases the main climate-related drivers influencing human exposure will be changes in the types of chemicals used by society, alterations in the amounts and patterns of chemical use, and changes in the rates of formation of natural toxins in natural systems. Also, GCC will affect the way in which human populations interact with the natural environment so as to alter the degree of exposure. Alongside changes in exposure, changes in the sensitivity of humans to chemical exposure are expected due to factors such as increases in the levels of heat stress, psychosocial factors, suppression of the immune system, and alterations in nutritional status due to changes in diet and the quality of foodstuffs.

While each individual impact of GCC on chemical exposure and human sensitivity may not be highly significant and may occur in either a positive or a negative direction, the potential cumulative impacts of multiple influences could significantly alter risks to human health. Despite significant uncertainties, the preponderance of the current evidence suggests that many human health risks from chemicals may be increased in the future if a business-as-usual approach is adopted. Changes in risks are likely to be most significant for chemicals where microbes, plants, and lower animals are involved in the source-to-receptor pathway. Increases in risks are likely to be seen for the natural toxins that are produced by microbes, algae, and plants. The risks of chemicals, such as pesticides, whose use is determined by population responses of fungi, weeds, and lower-order animals, or those, like mercury, whose speciation is altered by the activity of micro-organisms, will also be significantly affected. While the absolute magnitude of changes in average air concentrations related to GCC may be relatively small for a given air pollutant, widespread human exposures, increased peak concentrations in certain geographical areas, and significant health consequences make such impacts of GCC very important from a public-health standpoint.

These alterations in risks have implications for national and international decision makers involved in the regulation and authorization of chemical products and the monitoring and management of chemicals in environmental matrices and foodstuffs. The expected changes reveal that some of the scenarios and models currently used in health risk assessment of chemicals will need updates and revision in order to reflect some of the future changes described earlier. Monitoring methodologies may also need to be adapted in order to cope with increased variability in exposure, sensitivity, and risk, both spatially and temporally. Monitoring and sampling should be done at a frequency sufficient to capture variability, which is likely to increase in many places.

It is also important to recognize that human exposure to chemicals in the environment in the future will be affected by other, non-climate-related drivers such as increased urbanization, future technological developments (such as a move toward more environmentally benign pesticides), ongoing strategies to reduce emissions and other environmental releases (e.g., of persistent organic pollutants), and economic changes. In some cases, these drivers may have a bigger impact on human exposure (either increasing or lowering exposures) and risks than GCC alone.

There are, however, major gaps in our current understanding of how chemical risks will change. A concerted effort is therefore needed at an international level to better characterize the potential impacts of GCC and other future drivers on exposure, sensitivity, and risk. We recommend that work should focus on the following areas. First, the development of future models and scenarios of land use and social, technological, and economic change in order to provide a basis for informing how inputs of chemicals to the environment, in different regions of the world, may change in the future. Second, work should focus on the generation of improved data sets and models for determining future human exposure to chemicals in different environmental matrices. This work should consider the importance of emerging exposure pathways, such as increased inhalation of contaminated dust or exposure consequences of flooding, and consider the implications of human behavioral change on the degree of exposure. Furthermore, there should be focus the development of research programs that aim to fill gaps in our understanding of the interactions between climate and weather parameters and human sensitivity to chemical exposures. Focus should also be given to the refinement of regulatory models and procedures in the light of knowledge gained from work on exposure and human sensitivity to toxicants. Existing risk assessments and chemical management programs should also be updated to determine whether the risks of a current-use product could change in the future. Finally, work should give focus to the development of targeted surveillance schemes for the presence and health effects of select chemicals in different environmental compartments for different regions of the world and at smaller geographical scales to address inequities at the community level.

To address these knowledge gaps, input is required from a wide range of disciplines (including climate science, toxicology, exposure science, public health, environmental modeling, social science, economics, and environmental chemistry) and a range of sectors across the globe. It is essential that future research and assessment programs take a holistic approach and do not just focus on GCC-driven changes alone. Research programs should also include elements of technology transfer and capacity building for developing countries struggling to adapt to GCC impacts, many of which will be most vulnerable to the forecast changes in chemical exposure.
